# Self-Reported Hearing Impairment and Edentulism Trajectories and Multimorbidity in Chinese Middle-Aged and Older Adults: Associations Replicated in Chinese and US National Cohorts

**DOI:** 10.3390/healthcare14213802

**Published:** 2026-11-06

**Authors:** Maoyu Jiang, Yehai Liu

**Affiliations:** Department of Otolaryngology-Head and Neck Surgery, The First Affiliated Hospital of Anhui Medical University, Hefei 230022, China; 2346010195@stu.ahmu.edu.cn

**Keywords:** self-reported hearing impairment, edentulism, multimorbidity, latent class linear mixed model, CHARLS, HRS

## Abstract

Background and Objectives: Self-reported hearing impairment and edentulism are common age-related conditions, yet whether they act as independent markers of accumulating chronic disease multimorbidity remains uncertain. This study characterized their population trajectories and examined their associations with multimorbidity, testing cross-national reproducibility. Materials and Methods: Using four waves (2011–2018) of the China Health and Retirement Longitudinal Study (CHARLS) with external validation in the US Health and Retirement Study (HRS), we characterized self-reported hearing impairment and edentulism trajectories with latent class linear mixed models among 17,585 participants and, in 15,842 Wave 1 participants, examined associations with chronic disease multimorbidity after extensive covariate adjustment. Results: Self-reported hearing impairment prevalence rose from 54.6% to 66.4% across waves; edentulism ranged from 4.5% to 8.4%. A single-class model best fitted both outcomes, with significant per-wave increases in self-reported hearing impairment (0.051 per wave). Self-reported hearing impairment was associated with higher odds of two or more (OR = 1.42, 95% CI 1.31–1.53) and three or more (OR = 1.43, 95% CI 1.28–1.60) chronic conditions, replicated in Wave 4 (OR = 1.40 and 1.50) and in HRS (OR = 1.38). In a prospective analysis of participants free of multimorbidity at baseline, self-reported hearing impairment predicted incident multimorbidity at Wave 4 (OR = 1.35 in the age- and sex-adjusted model; OR = 1.28 in the fully adjusted model). By contrast, edentulism showed no consistent independent association with multimorbidity; its crude association was explained by age. Conclusions: A single self-reported hearing question is a sensitive, dose-dependent, and cross-nationally reproducible indicator of coexisting multiple diseases in middle-aged and older adults and merits consideration as a low-cost first-line indicator in primary care. Edentulism was not independently associated with multimorbidity after accounting for age.

## 1. Introduction

Self-reported hearing impairment and edentulism are common age-related conditions that impose a substantial health burden in aging populations. Hearing loss is among the leading causes of years lived with disability, and the World Health Organization estimates that more than 1.5 billion people live with some degree of hearing impairment [1,2]. Tooth loss and edentulism (complete tooth loss) similarly affect a substantial share of older populations, including an estimated ≈8% of community-dwelling Chinese adults aged 60 years and older [3]. In China, more than half of middle-aged and older adults report some degree of hearing difficulty, underscoring the scale of both conditions in this rapidly aging population [4,5].

Both conditions have been individually linked to broader health outcomes. Hearing loss is a recognized modifiable risk factor for dementia and has been associated with reduced social engagement and poorer health [4,6,7], while edentulism has been associated with impaired nutrition and reduced quality of life [8,9] and remains a substantial oral-health challenge in aging populations [10]. Multimorbidity is increasingly recognized as a major driver of disability, healthcare use, and mortality in older adults [11], and China is aging rapidly, with multimorbidity affecting a large and growing share of its older population [5]. Both conditions plausibly share systemic pathways with chronic disease, including chronic inflammation, vascular injury, and nutritional compromise, providing a biological rationale for their potential association with multimorbidity [6,12,13]. Yet whether these sensory and oral impairments act as independent or early markers of accumulating chronic disease multimorbidity—the coexistence of two or more chronic conditions—remains uncertain [11,14,15,16].

To date, few studies have characterized the population-level trajectories of self-reported hearing impairment and edentulism jointly, and the evidence linking each condition to multimorbidity remains largely cross-sectional and confined to single cohorts [11,17]. Most prior work has examined self-reported hearing impairment and edentulism separately, seldom accounting for their mutual confounding, and few have tested whether such associations replicate across independent, nationally representative populations. Understanding whether these conditions follow distinct trajectories and whether one or both are associated with multimorbidity risk could guide targeted screening and prevention in primary care.

We therefore used four waves (2011–2018) of the nationally representative China Health and Retirement Longitudinal Study (CHARLS) to (i) characterize the trajectories of self-reported hearing impairment and edentulism with latent class linear mixed models (LCMM) [18,19], and (ii) examine their associations with chronic disease multimorbidity, replicating the primary hearing impairment–multimorbidity finding internally (Wave 4) and externally in the US Health and Retirement Study (HRS) [20] and across individual chronic conditions.

Beyond replicating established associations, this study contributes to the field in four ways. First, it jointly characterizes four-wave (2011–2018) population trajectories of self-reported hearing impairment and edentulism in a nationally representative Chinese cohort, extending prior single-condition, largely cross-sectional work. Second, it adds a prospective analysis showing that baseline hearing impairment predicts incident multimorbidity, thereby addressing reverse causation, which a cross-sectional design alone cannot exclude. Third, it examines a sensory (hearing) and an oral (edentulism) marker within the same models, allowing their independent contributions to be compared directly. Fourth, it replicates the primary association in an independent US cohort (HRS) using an identical analytical pipeline, supporting cross-national generalizability. Together, these features indicate that a single self-reported hearing question can serve as a sensitive, dose-dependent, low-cost indicator of coexisting multiple diseases, with potential value for multimorbidity assessment in resource-limited primary-care settings.

## 2. Materials and Methods

### 2.1. Study Design and Participants

This was a population-based cohort analysis of CHARLS, a nationally representative longitudinal survey of Chinese adults aged 45 years and older (by design, also including 40–44-year-old refresher respondents) [18]. Four waves (2011, 2013, 2015, 2018) were used, yielding 25,457 unique respondents. Participants with complete exposure data were included in the trajectory analysis (*N* = 17,585, contributing person-wave observations across all four waves); the Wave 1 cross-sectional analytic sample comprised 15,842 participants after excluding those with missing covariates (Supplementary Table S1 and Supplementary Table S2; Supplementary Figure S1). Thus, the trajectory and cross-sectional samples serve different analytic purposes: the former characterizes within-person change over time, whereas the latter tests cross-sectional exposure–outcome associations at baseline. The US Health and Retirement Study (HRS), a nationally representative US cohort [21], was used for external validation.

### 2.2. Variables

Hearing impairment was defined by a self-rated five-point hearing scale (1 = excellent to 5 = poor); scores of 4–5 (fair or poor) were classified as hearing impairment, equivalent to a binary none/mild versus moderate/severe classification. Edentulism was defined as complete tooth loss (self-reported no natural teeth). Multimorbidity was defined as having two or more (and, in secondary analyses, three or more) of nine chronic conditions: hypertension, dyslipidemia, diabetes, cancer, chronic lung disease, kidney disease, heart disease, stroke, and arthritis. Covariates included age, sex, education, urban/rural residence, depressive symptoms (CESD-10), and activities of daily living (ADL) disability.

### 2.3. Statistical Analysis

Latent class linear mixed models with one to five latent classes were fitted using the lcmm package (version 2.2.2) in R 4.4.3 (R Foundation for Statistical Computing, Vienna, Austria) [19], with convergence defined by a relative log-likelihood change below 1 × 10^−3^ and a maximum of 1000 iterations; the optimal number of classes was selected by the Bayesian Information Criterion (BIC). Cross-sectional associations were estimated with multivariable logistic regression and cluster-robust standard errors (sandwich HC0), adjusting for all covariates; two-sided Wald tests with alpha 0.05 were used, and actual *p*-values are reported. Exploratory analyses were repeated for each individual chronic disease using pooled data across the four waves (Supplementary Table S3) [17]. For the edentulism–multimorbidity association, we assessed age-confounding by progressive adjustment, age-stratified analyses, and an edentulism-by-age interaction. Sensitivity analyses included a Wave 4 replication (Supplementary Table S4), a two-class trajectory specification (Supplementary Table S5; Supplementary Figure S2), a restricted cubic spline dose–response analysis with three knots at the 10th, 50th, and 90th percentiles of the hearing score (Supplementary Figure S3), and exclusion of cancer cases. A Poisson count model estimated the rate ratio in HRS [22]. Although the latent class framework was fitted with one to five latent classes a priori, a single-class solution was ultimately selected; a one-class result indicates that a single population trajectory describes the data adequately and does not invalidate the longitudinal modeling approach, as the framework remains informative about the overall shape and variability of the population trajectories over time.

In addition, a prospective analysis was performed to complement the cross-sectional findings: participants free of multimorbidity at Wave 1 (<2 of the nine chronic conditions) were followed to Wave 4, and incident multimorbidity (new occurrence of ≥2 and ≥3 conditions) was regressed on baseline hearing impairment using the same sequential models (Model 1: age and sex; Model 2: additionally education and urban/rural residence; Model 3: additionally CESD-10 and ADL disability) with cluster-robust standard errors (Supplementary Table S6). CESD-10 and ADL disability were treated as potential mediators in this framework; Model 2, which excludes them, is presented alongside Model 3 to show which adjustments weaken the association. A discrete-time survival analysis across Waves 2–4 was not feasible because the Wave 2 and Wave 3 chronic disease modules were administered only to newly enrolled respondents (14.9% and 2.5% coverage, respectively) and capture incident diagnoses only, without carry-forward variables, precluding reconstruction of interim disease burden. Supplementary Table S7 documents the harmonization of the chronic disease list, questionnaire items, response coding, diagnostic definitions, and carry-forward method between CHARLS and HRS. Statistical analyses were performed using R 4.4.3 (https://www.r-project.org/ accessed on 27 October 2026; The R Foundation) and Free Statistics software version 2.6 (https://app.clinicalscientists.cn/ accessed on 27 October 2026).

### 2.4. Ethics

This study used de-identified public-use data from the China Health and Retirement Longitudinal Study (CHARLS) and the Health and Retirement Study (HRS). All participants provided written informed consent in the original surveys, and the ethics approvals for both cohorts are detailed in the Institutional Review Board Statement. Because all analyses used de-identified public-use data, no additional ethics approval was required. The manuscript was prepared in accordance with the Strengthening the Reporting of Observational Studies in Epidemiology (STROBE) guideline [23].

## 3. Results

### 3.1. Prevalence and Trajectories

**Figure 1:**
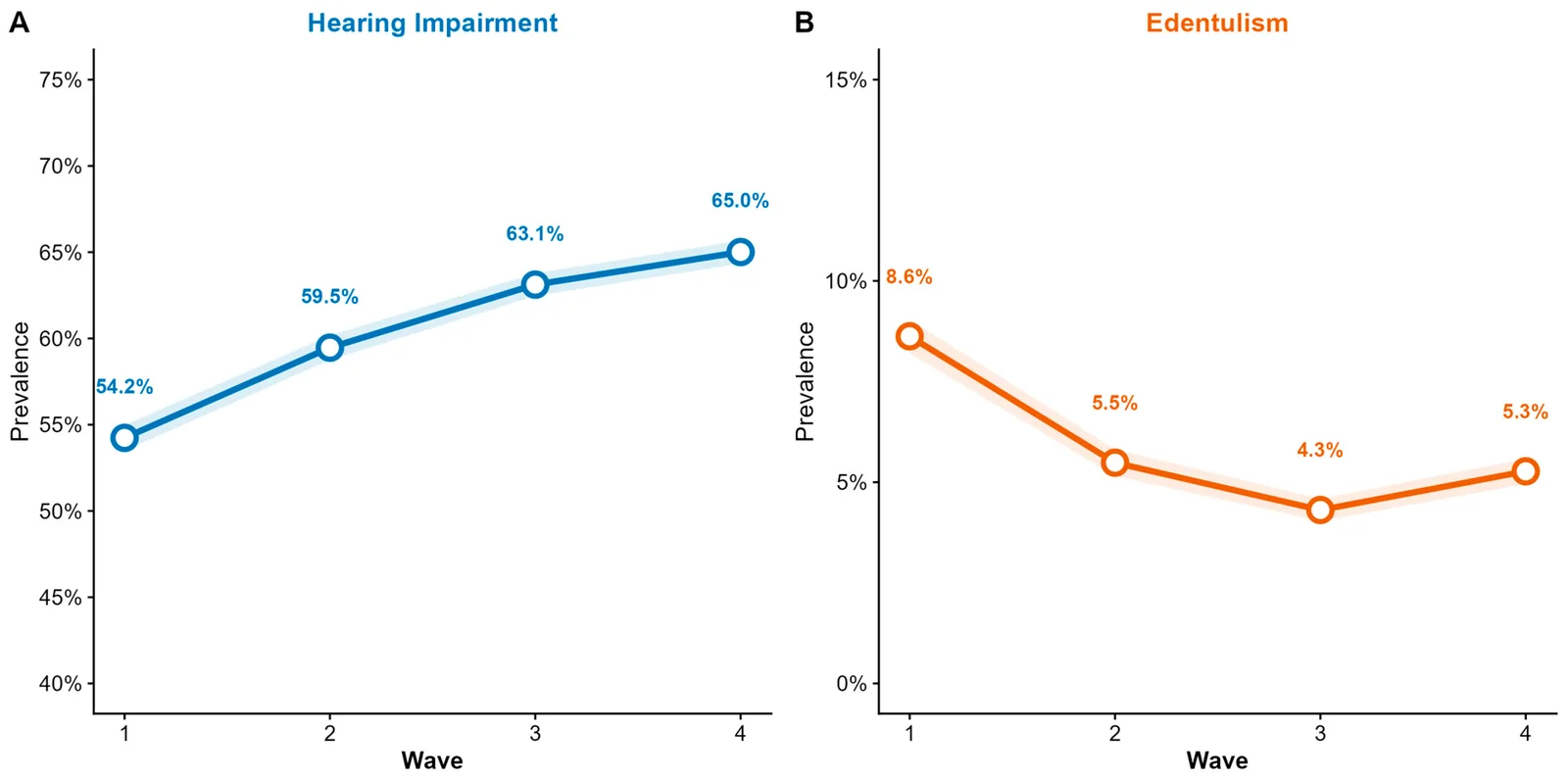
Population-level prevalence trajectories of hearing impairment (**A**) and edentulism (**B**) across the four CHARLS waves (2011–2018). Points represent observed prevalence per wave; shaded ribbons represent 95% confidence intervals of the observed prevalence. CI, confidence interval.

**Figure 2:**
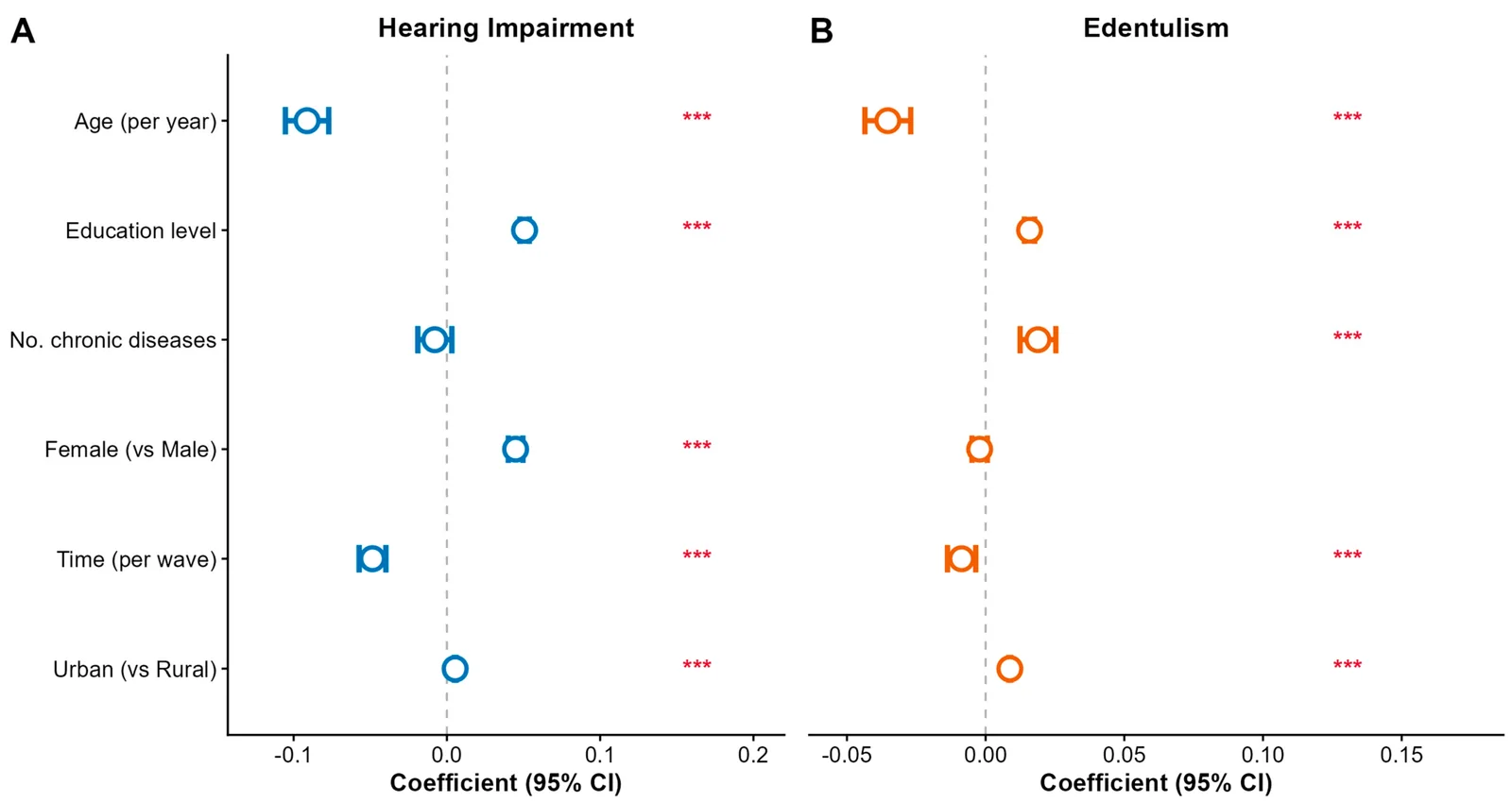
Latent class linear mixed model (LCMM) coefficient estimates for (**A**) hearing impairment and (**B**) edentulism trajectories. Circles represent point estimates; horizontal bars represent 95% confidence intervals. Time is expressed per survey wave. CI, confidence interval; *** *p* < 0.001.

Baseline characteristics are shown in Table 1. Self-reported hearing impairment prevalence increased from 54.6% at Wave 1 to 66.4% at Wave 4, whereas edentulism prevalence ranged from 4.5% to 8.4% (Figure 1). A single-class LCMM best-fitted both outcomes (Table 2; Figure 2; hearing BIC = 65,872; edentulism BIC = −3736), with models of two to five classes failing to converge (singular Hessian or non-positive-definite variance–covariance matrices across repeated random-start specifications). The time coefficient was 0.051 per wave (*p* < 0.001) for self-reported hearing impairment and 0.016 per wave (*p* < 0.001) for edentulism (Table 2). The positive time coefficient for edentulism reflects the within-person, age-adjusted increase in edentulism propensity with advancing age; it does not imply a rising crude prevalence, which in fact declined across waves (8.4% at Wave 1 to 4.5% at Wave 3) as the trajectory sample became younger on average through refreshment and attrition (Figure 1).

### 3.2. Self-Reported Hearing Impairment and Multimorbidity

**Figure 3:**
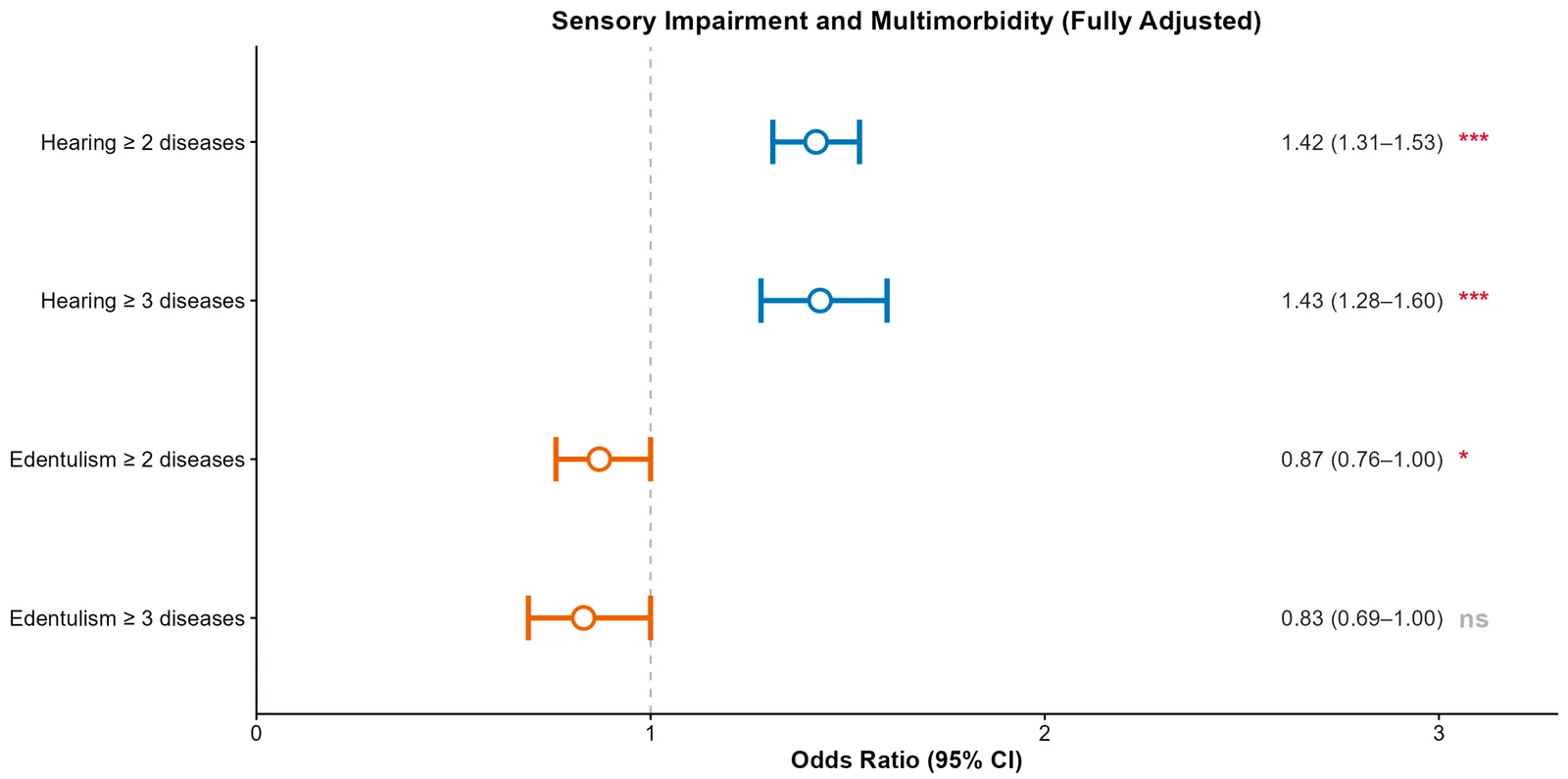
Odds ratios (95% CI) for the association between hearing impairment and edentulism with chronic disease multimorbidity (two or more, and three or more conditions) in fully adjusted models. Models were adjusted for age, sex, education, urban/rural residence, CESD-10, and ADL disability. Circles represent point estimates; horizontal bars represent 95% confidence intervals. * *p* < 0.05, *** *p* < 0.001; ns, not significant.

**Figure 4:**
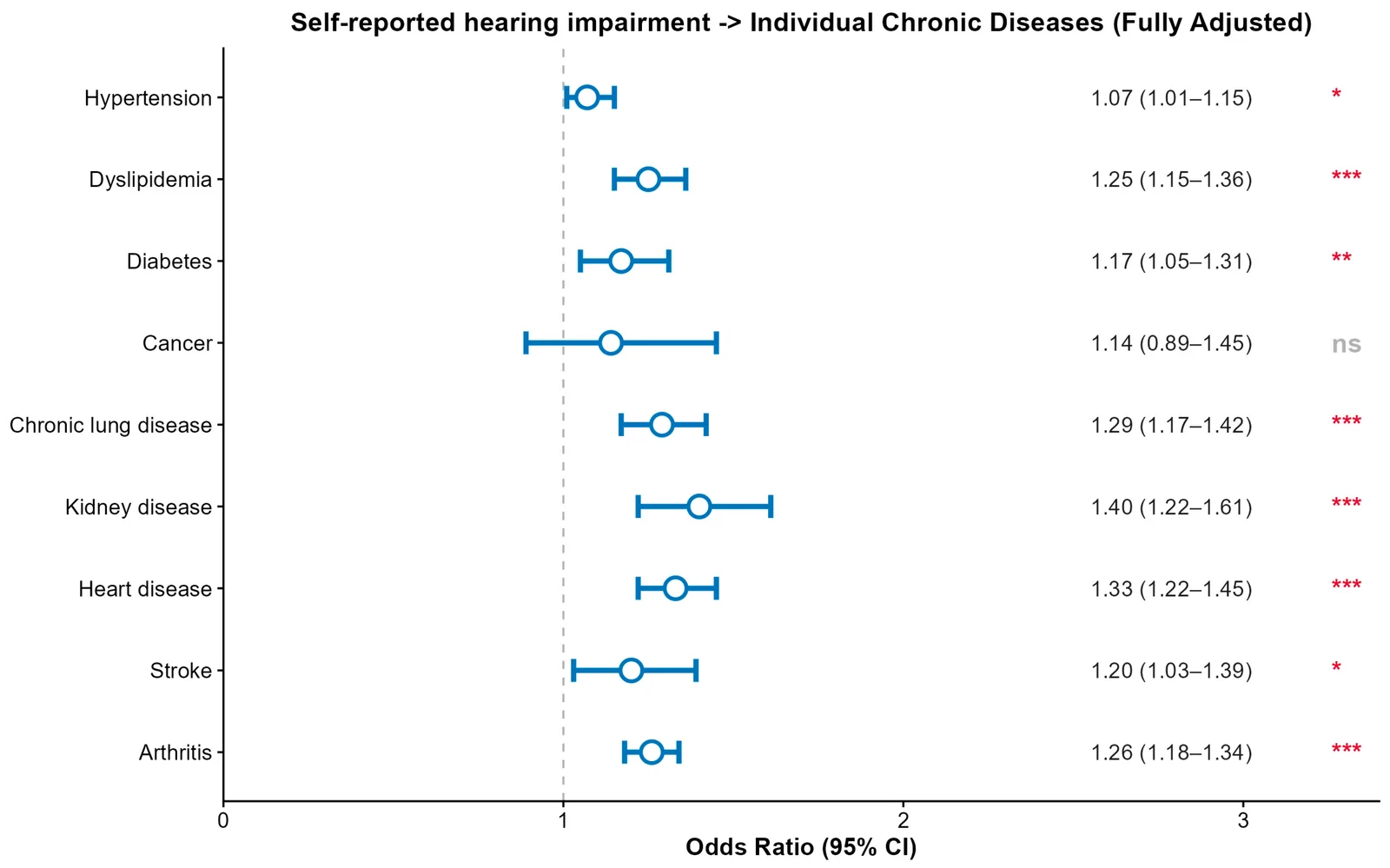
Odds ratios (95% CI) for the exploratory association between hearing impairment and each of the nine individual chronic diseases in fully adjusted models. Models were adjusted for age, sex, education, urban/rural residence, CESD-10, and ADL disability. Circles represent point estimates; horizontal bars represent 95% confidence intervals. * *p* < 0.05, ** *p* < 0.01, *** *p* < 0.001; ns, not significant.

In fully adjusted models, self-reported hearing impairment was positively associated with multimorbidity (two or more conditions: OR = 1.42, 95% CI 1.31–1.53; three or more: OR = 1.43, 95% CI 1.28–1.60) (Table 3; Figure 3). A dose–response relationship was observed across the five-point self-rated hearing scale (Supplementary Figure S3; predicted probability 15.2% to 23.6%) [17]. In exploratory pooled individual-disease analyses, self-reported hearing impairment was positively associated with hypertension (OR = 1.07, 95% CI 1.01–1.15), chronic lung disease (OR = 1.29, 95% CI 1.17–1.42), kidney disease (OR = 1.40, 95% CI 1.22–1.61), and arthritis (OR = 1.26, 95% CI 1.18–1.34), with similar positive associations across the remaining conditions (Figure 4; Supplementary Table S3).

### 3.3. Edentulism and Multimorbidity

**Figure 5:**
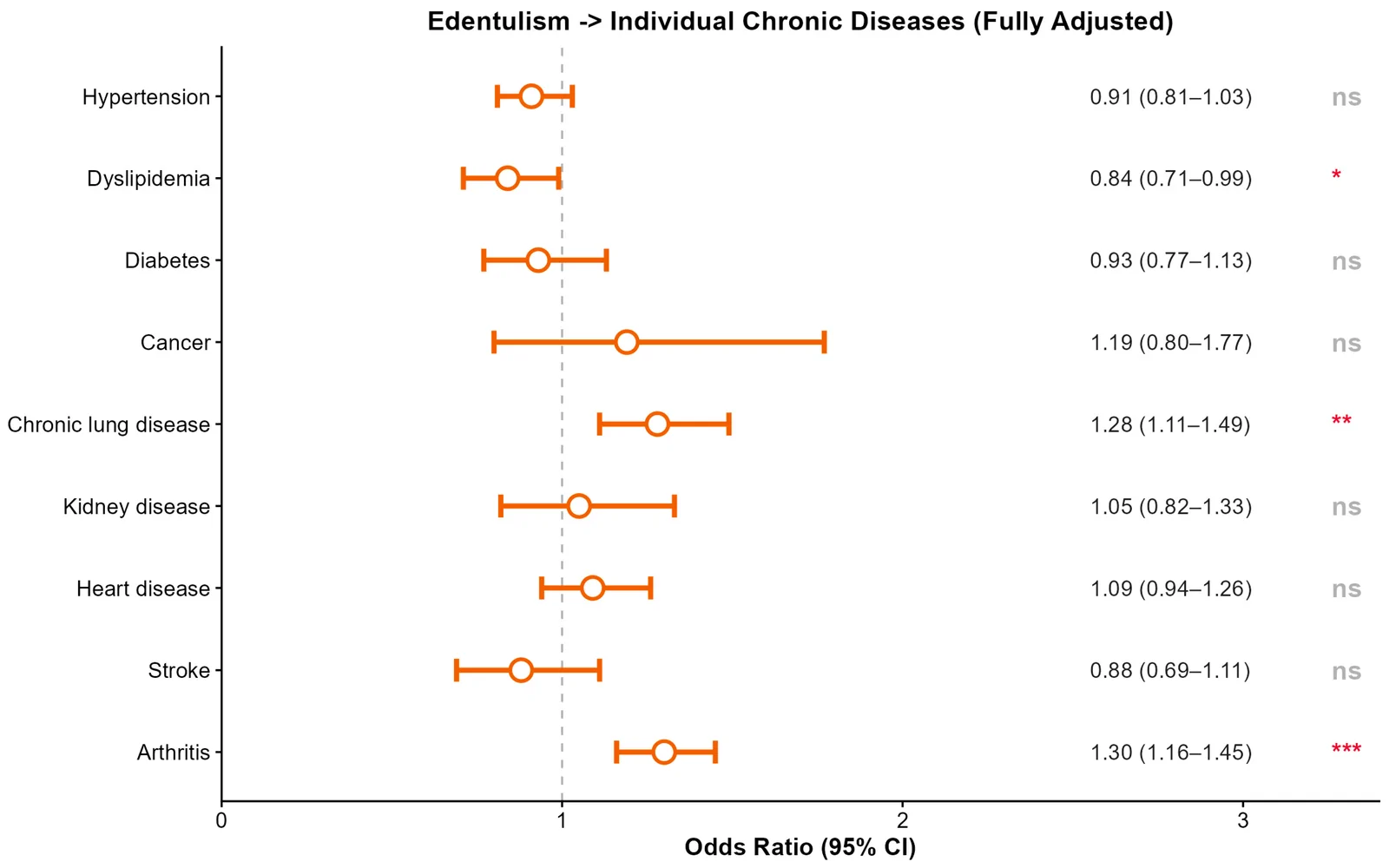
Odds ratios (95% CI) for the association between edentulism and each of the nine individual chronic diseases in fully adjusted models. Models were adjusted for age, sex, education, urban/rural residence, CESD-10, and ADL disability. Circles represent point estimates; horizontal bars represent 95% confidence intervals. * *p* < 0.05, ** *p* < 0.01, *** *p* < 0.001; ns, not significant.

Edentulism showed no consistent independent association with multimorbidity. Crudely, edentulism was positively associated with multimorbidity (OR = 1.37, 95% CI 1.22–1.55); this was markedly attenuated and no longer indicated a positive association after adjustment, with the estimate borderline and in the protective direction (OR = 0.87, 95% CI 0.76–1.00, *p* = 0.048). Within each age stratum, no significant association was evident, and the edentulism-by-age interaction was significant (*p* < 0.001). Individual-condition analyses were heterogeneous, with no significant association for hypertension (OR = 0.91, 95% CI 0.81–1.03) and positive associations for chronic lung disease (OR = 1.28, 95% CI 1.11–1.49) and arthritis (OR = 1.30, 95% CI 1.16–1.45) (Figure 5; Supplementary Table S3).

### 3.4. External Validation and Replication

In HRS (*N* = 60,525 observations from 22,890 adults), self-reported hearing impairment was associated with higher odds of two or more (OR = 1.38, 95% CI 1.30–1.47) and three or more (OR = 1.36, 95% CI 1.28–1.45) chronic conditions, with a consistent Poisson rate ratio (RR = 1.106, 95% CI 1.089–1.125) (Supplementary Figure S4; Supplementary Table S8). The association was replicated in Wave 4 (OR = 1.40 and 1.50, respectively; Supplementary Table S4), where chronic disease status for previously diagnosed respondents was carried forward using the official CHARLS carry-forward variables.

### 3.5. Prospective Analysis of Incident Multimorbidity

Among the 9936 Wave 1 participants free of multimorbidity at baseline, self-reported hearing impairment was associated with incident multimorbidity at Wave 4 (two or more conditions: OR = 1.35, 95% CI 1.24–1.47, in Model 1; OR = 1.36, 95% CI 1.25–1.48, in Model 2; OR = 1.28, 95% CI 1.17–1.39, in Model 3). Results were similar for incident multimorbidity of three or more conditions and in the disease-free baseline subsample (*N* = 5576; Model 3 OR = 1.30–1.41), all *p* < 0.001 (Supplementary Table S6). The modest attenuation from Model 2 (OR 1.36) to Model 3 (OR 1.28) after adding CESD-10 and ADL disability is consistent with partial mediation via the hearing impairment → depression → disability pathway, indicating that hearing impairment precedes disease accumulation rather than merely co-occurring with it. Edentulism showed no independent association with incident multimorbidity (fully adjusted OR = 0.95, 95% CI 0.79–1.13).

## 4. Discussion

In this large, nationally representative cohort, self-reported hearing impairment followed a worsening population trajectory and was robustly associated with chronic disease multimorbidity in a dose-dependent manner, with the association confirmed across two independent national cohorts (CHARLS, China and HRS, USA) [17]. Multimorbidity is highly prevalent in Chinese older adults [5], and identifying an accessible early indicator of accumulating multimorbidity burden is therefore of direct public-health relevance. These results position self-reported hearing impairment as a low-cost screening prompt aligned with WHO hearing-priority strategies [1,2,22] and with evidence that hearing screening is feasible in primary care [24]. Because hearing status is elicited in seconds without equipment, it is readily scalable to primary care consultations, community-based health examinations, and resource-limited settings where objective audiometry is unavailable; a positive finding would prompt a multimorbidity risk assessment rather than replace clinical judgement. These findings should not, however, be interpreted as validating a formal screening instrument; establishing clinical action thresholds (for example, whether a ‘fair or poor’ rating should trigger audiometric referral or broader health assessment) and quantifying sensitivity, specificity, and predictive values against audiometric gold standards require dedicated implementation research. To our knowledge, this cross-national replication across two independent, nationally representative cohorts (China and the USA) provides unusually robust evidence that the association is generalizable rather than population-specific. In contrast to recent cross-sectional Chinese studies of hearing impairment and cardiometabolic multimorbidity [17], the present study adds four-wave trajectory modeling, a prospective analysis of incident multimorbidity, and parallel external replication in HRS, and it jointly examines hearing impairment and edentulism. By contrast, as detailed below, edentulism showed a different pattern and was not an independent indicator of multimorbidity.

The cross-national reproducibility of the hearing impairment–multimorbidity association strengthens its generalizability and is consistent with prior evidence linking hearing impairment to systemic chronic disease [6,7,17,21]. Several mechanisms may explain the association. Midlife hypertension contributes to cochlear microvascular damage and age-related hearing decline [12], and shared vascular risk factors (hypertension, dyslipidemia) plausibly underlie both self-reported hearing impairment and cardiovascular multimorbidity. Self-reported hearing impairment may also reduce social engagement and communication, lowering preventive healthcare contact and allowing chronic conditions to accumulate undetected [25]. Consistent with this reasoning, self-reported hearing difficulty likely captures a broader construct than pure cochlear pathology alone, integrating the functional, psychosocial, and help-seeking aspects of hearing difficulty in older adults. These pathways are not mutually exclusive, and the present cross-sectional design cannot separate them; nonetheless, the graded dose–response pattern is more consistent with an aggregate multimorbidity effect than with a single-disease artifact, although this cannot be definitively established from cross-sectional data. Together, these vascular and psychosocial pathways plausibly underlie the observed dose–response association. As a sensitivity check on shared vascular pathways, we re-fitted the primary hearing impairment–multimorbidity model with hypertension additionally included as a covariate; the association was essentially unchanged, indicating that the finding was not attributable to mutual adjustment for hypertension.

By contrast, edentulism was not independently associated with multimorbidity after accounting for age. This crude positive association was explained by the older age of edentulous participants, and no association persisted within any age stratum, with a significant edentulism-by-age interaction. Although tooth loss has been linked to cardiovascular disease and mortality in some cohorts [26,27,28], and edentulism itself predicts all-cause mortality in China [27], the apparent inverse signal observed here was attributable to age confounding; a pattern consistent with selective survival cannot be excluded, although the present study was not designed to test this directly. Early-life adversity shapes edentulism in Chinese adults [3], and the oral-systemic relevance of retained teeth and dental caries has been reported [13]; we therefore caution that the present null finding does not exclude broader oral-systemic effects—but edentulism alone does not appear to be an informative independent indicator of multimorbidity. This negative finding is scientifically useful: it helps distinguish which common age-related sensory and oral conditions carry independent multimorbidity information.

The present findings align with evidence that early-life and socioeconomic exposures shape later-life chronic disease [29] and with longitudinal work on cognitive and frailty trajectories in Chinese older adults [30], reinforcing the importance of population-level trajectory approaches for aging-related conditions. Strengths of this study include the large national sample (25,457 unique respondents), longitudinal trajectory methods, comprehensive covariate adjustment with cluster-robust standard errors, dose–response analysis, honest treatment of the edentulism null finding, and external validation in an independent US cohort.

Results were stable across a range of sensitivity and subgroup analyses (Supplementary Figure S5; Supplementary Table S9), and a supplementary analysis of single versus dual sensory impairment showed stepwise higher multimorbidity odds with increasing sensory impairment (Supplementary Figure S6). Several limitations warrant consideration. First, both hearing impairment and edentulism were self-reported rather than objectively measured (by audiometry or dental examination). This misclassification is likely to be non-differential with respect to the outcome, so it would bias estimates toward the null, and the true associations may be stronger than reported. Second, the main association analyses were cross-sectional and cannot establish causality. We partly addressed this by adding a prospective analysis showing that hearing impairment precedes incident multimorbidity, but residual confounding cannot be excluded because data on smoking, noise exposure, diet, and access to healthcare were unavailable. Third, multimorbidity was defined as a simple count of chronic conditions, which does not capture disease severity, treatment burden, or how well conditions are controlled. Fourth, the individual-disease analyses were exploratory and were not corrected for multiple comparisons. Fifth, the HRS replication covered fewer shared conditions than CHARLS, and neither cohort provided objective sensory or oral measures. Sixth, CHARLS does not distinguish conductive, sensorineural, or mixed hearing loss, so the underlying mechanism cannot be resolved. Future studies with audiometric phenotyping and structural equation models—linking socioeconomic, lifestyle, and health factors—would help clarify these pathways.

Taken together, our findings support self-reported hearing impairment as a cross-nationally reproducible indicator of coexisting multiple diseases, whereas edentulism did not show an independent association.

## 5. Conclusions

Self-reported hearing impairment is a sensitive, dose-dependent, and cross-nationally reproducible indicator of coexisting multiple diseases in middle-aged and older adults and merits consideration as a low-cost first-line indicator in primary care. Edentulism was not independently associated with multimorbidity after accounting for age, and its crude association reflected age confounding. Prospective studies with objective sensory and oral assessment are warranted to confirm these findings.

## Figures and Tables

**Table 1:** Baseline Characteristics of the Wave 1 Analytic Sample, Overall and by Hearing Status (*N* = 15,842).

Characteristic	Overall	Non-Hearing-Impaired	Hearing-Impaired	*p*-Value
Age, years	59.0 ± 9.9	57.1 ± 9.4	60.5 ± 10.1	<0.001
Sex, *n* (%)				0.062
Male	7432 (46.9)	3447 (47.7)	3985 (46.2)	
Female	8410 (53.1)	3775 (52.3)	4635 (53.8)	
Education, *n* (%)				<0.001
Primary or less	10,574 (66.7)	4360 (60.4)	6214 (72.1)	
Middle school	3270 (20.6)	1675 (23.2)	1595 (18.5)	
High school or above	1998 (12.6)	1187 (16.4)	811 (9.4)	
Residence, *n* (%)				<0.001
Urban	3575 (22.6)	1890 (26.2)	1685 (19.5)	
Rural	12,267 (77.4)	5332 (73.8)	6935 (80.5)	
Edentulism, *n* (%)				<0.001
No	14,518 (91.6)	6790 (94.0)	7728 (89.7)	
Yes	1324 (8.4)	432 (6.0)	892 (10.3)	
No. of chronic diseases	1.1 ± 1.2	0.9 ± 1.1	1.2 ± 1.2	<0.001
Multimorbidity (≥2), *n* (%)				<0.001
No	11,410 (72.0)	5643 (78.1)	5767 (66.9)	
Yes	4432 (28.0)	1579 (21.9)	2853 (33.1)	
Multimorbidity (≥3), *n* (%)				<0.001
No	14,038 (88.6)	6625 (91.7)	7413 (86.0)	
Yes	1804 (11.4)	597 (8.3)	1207 (14.0)	

Values are mean ± SD for continuous variables and *n* (%) for categorical variables. Hearing impairment was defined as a self-rated hearing score of 4–5 (fair or poor). *p*-values are from two-sample *t*-tests (continuous variables) or chi-squared tests (categorical variables) comparing hearing-impaired with non-hearing-impaired participants.

**Table 2:** Joint LCMM Trajectory Model Estimates.

Parameter	Hearing: Coef (SE)	Hearing: *p*	Edentulism: Coef (SE)	Edentulism: *p*
Age (per year)	0.005 (0.000)	<0.001	0.009 (0.000)	<0.001
Cholesky 1	0.266 (0.003)	<0.001	0.170 (0.002)	<0.001
Cholesky 2	0.016 (0.006)	0.013	0.017 (0.003)	<0.001
Education (per level)	−0.049 (0.004)	<0.001	−0.009 (0.003)	<0.001
Intercept	0.327 (0.024)	<0.001	−0.422 (0.014)	<0.001
Number of chronic diseases	0.045 (0.002)	<0.001	−0.002 (0.001)	0.129
Female (vs male)	−0.008 (0.006)	0.163	0.019 (0.003)	<0.001
Random-intercept SD (latent scale)	0.391 (0.002)	<0.001	0.190 (0.001)	<0.001
Time (per wave)	0.051 (0.002)	<0.001	0.016 (0.001)	<0.001
Urban (vs rural)	−0.091 (0.007)	<0.001	−0.035 (0.004)	<0.001

Abbreviations: LCMM, latent class linear mixed model; SE, standard error; *p*, *p*-value from a two-sided z-test. Coefficients are on the latent-process scale. Time is expressed per survey wave. The random-intercept SD is a standard deviation on the latent (log) scale and is therefore non-negative by definition; the original negative signs were a sign-convention artifact of the lcmm Cholesky parameterization and have been removed. Models were fitted with the lcmm package (version 2.2.2) in R 4.4.3.

**Table 3:** Association Between Self-Reported Hearing Impairment and Edentulism with Chronic Disease Multimorbidity (Model-by-Model).

Exposure	Outcome	Model	OR (95% CI)	*p*-Value
Hearing	>=2 chronic diseases	Model 0	1.77 (1.65–1.90)	<0.001
Hearing	>=2 chronic diseases	Model 1	1.60 (1.49–1.72)	<0.001
Hearing	>=2 chronic diseases	Model 2	1.42 (1.31–1.53)	<0.001
Hearing	>=3 chronic diseases	Model 0	1.81 (1.63–2.00)	<0.001
Hearing	>=3 chronic diseases	Model 1	1.63 (1.46–1.81)	<0.001
Hearing	>=3 chronic diseases	Model 2	1.43 (1.28–1.60)	<0.001
Edentulism	>=2 chronic diseases	Model 0	1.37 (1.22–1.55)	<0.001
Edentulism	>=2 chronic diseases	Model 1	0.92 (0.81–1.05)	0.228
Edentulism	>=2 chronic diseases	Model 2	0.87 (0.76–1.00)	0.048
Edentulism	>=3 chronic diseases	Model 0	1.31 (1.11–1.54)	0.001
Edentulism	>=3 chronic diseases	Model 1	0.88 (0.74–1.05)	0.159
Edentulism	>=3 chronic diseases	Model 2	0.83 (0.69–1.00)	0.055

Abbreviations: OR, odds ratio; CI, confidence interval. Model 0: unadjusted. Model 1: adjusted for age and sex. Model 2: fully adjusted. Standard errors were clustered at the individual level using a sandwich estimator (HC0).

## Data Availability

CHARLS data are publicly available at https://charls.pku.edu.cn/ and HRS data at https://hrs.isr.umich.edu/ after registration. Analysis scripts supporting this study are available at https://doi.org/10.6084/m9.figshare.33264594; they will be released following manuscript acceptance and are otherwise available from the corresponding author upon reasonable request.

## Supplementary Materials

The following supporting information can be downloaded at: https://www.mdpi.com/article/10.3390/healthcare14213802/s1, Figure S1: STROBE participant flow diagram; Figure S2: Two-class hearing impairment trajectories; Figure S3: Restricted cubic spline dose–response curve; Figure S4: HRS external validation; Figure S5: Sensitivity analysis caterpillar plot; Figure S6: Association between single versus dual sensory impairment and chronic disease; Table S1: Sample characteristics by CHARLS wave; Table S2: Missing data patterns; Table S3: Association between sensory/oral impairments and individual chronic diseases; Table S4: Wave 4 (2018) replication analysis; Table S5: LCMM trajectory heterogeneity sensitivity analysis; Table S6: Wave 1 self-reported hearing impairment and edentulism predicting incident multimorbidity at Wave 4; Table S7: Harmonization of chronic disease definitions between CHARLS and HRS; Table S8: HRS external validation; Table S9: Sensitivity and subgroup analyses.
