# Supplementary material for: Self-Reported Hearing Impairment and Edentulism Trajectories and Multimorbidity in Chinese Middle-Aged and Older Adults: Associations Replicated in Chinese and US National Cohorts
Source: Healthcare (Basel). 2026 Nov 6;14(21):3802. doi: 10.3390/healthcare14213802 (PMC13762499; doi:10.3390/healthcare14213802)
Supplement: Supplementary file 1 [file healthcare-14-03802-s001.zip › healthcare-4547939-supplementary.pdf]

healthcare

MDPI

Supplementary Information

Supplementary Figures

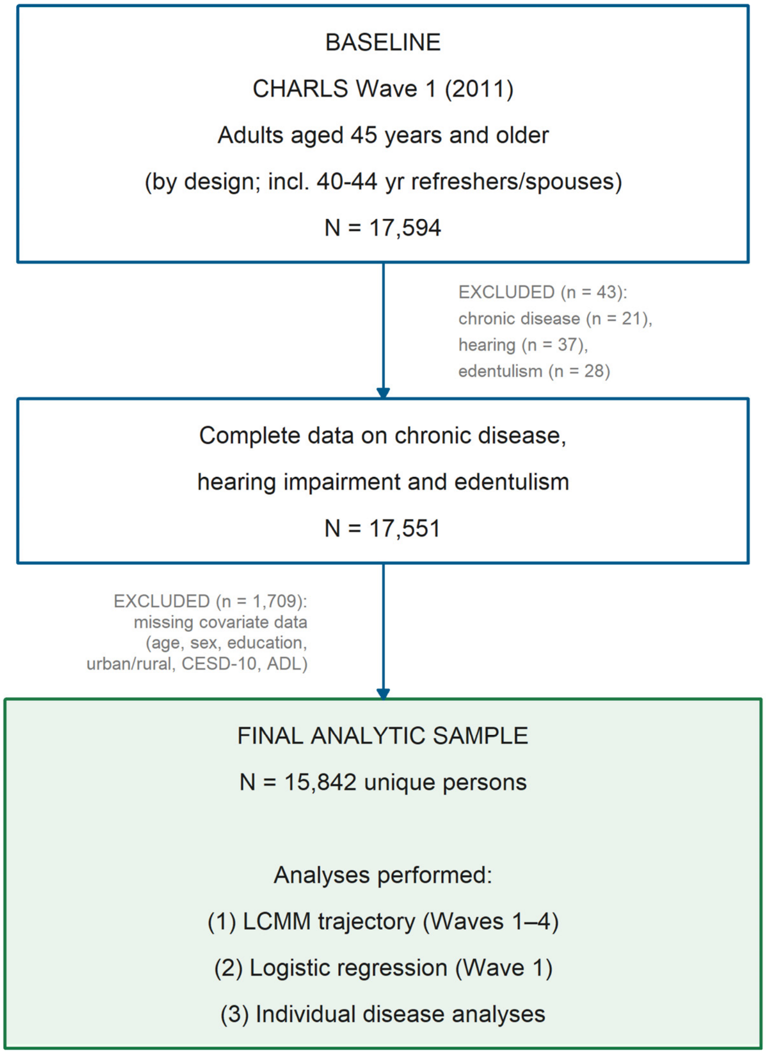

Supplementary Figure S1. STROBE participant flow diagram for CHARLS Wave 1. Of 17,594 Wave 1 participants, 43 were excluded for missing exposure data (37 with missing hearing impairment data, 28 with missing edentulism data, and 21 with missing chronic disease data; exclusions overlapped across variables), and a further 1,709 for missing other covariates (age, sex, education, urban/rural residence, CESD-10, ADL), yielding a complete analytic sample of 15,842 participants.

Waves 2-3 chronic disease items were administered exclusively to new respondents; Wave 4 (2018) replication used the official carry-forward variables.

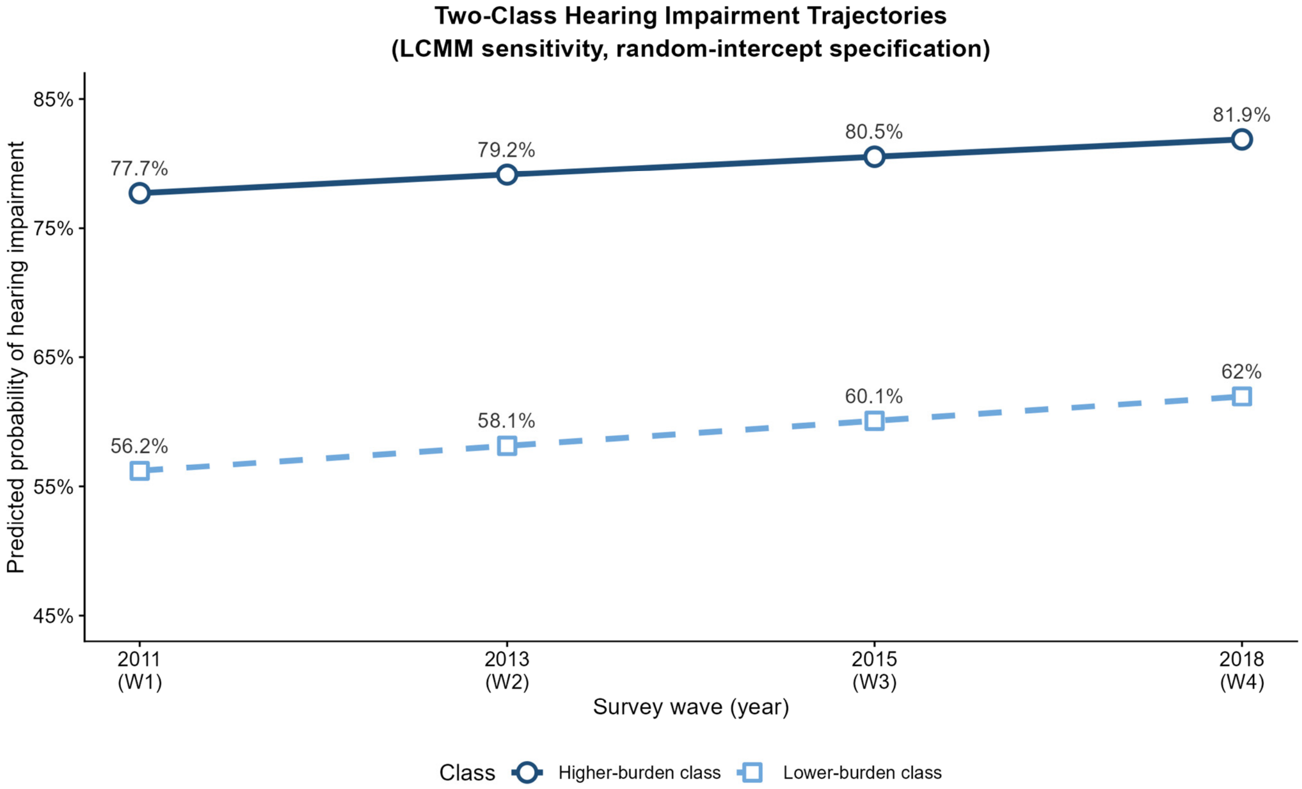

Supplementary Figure S2. Two-class hearing impairment trajectories from a random-intercept LCMM sensitivity analysis. Under the simplified random-intercept (fixed-slope) specification, a two-class solution converged for hearing impairment; classes were distinguished primarily by baseline burden (64.3% higher-burden class: predicted prevalence 73% at Wave 1 to 78% at Wave 4; 35.7% lower-burden class: 50% to 56%), sharing a similar rate of increase. The primary analysis retained the random-slope single-class model as the more conservative population-average estimate.

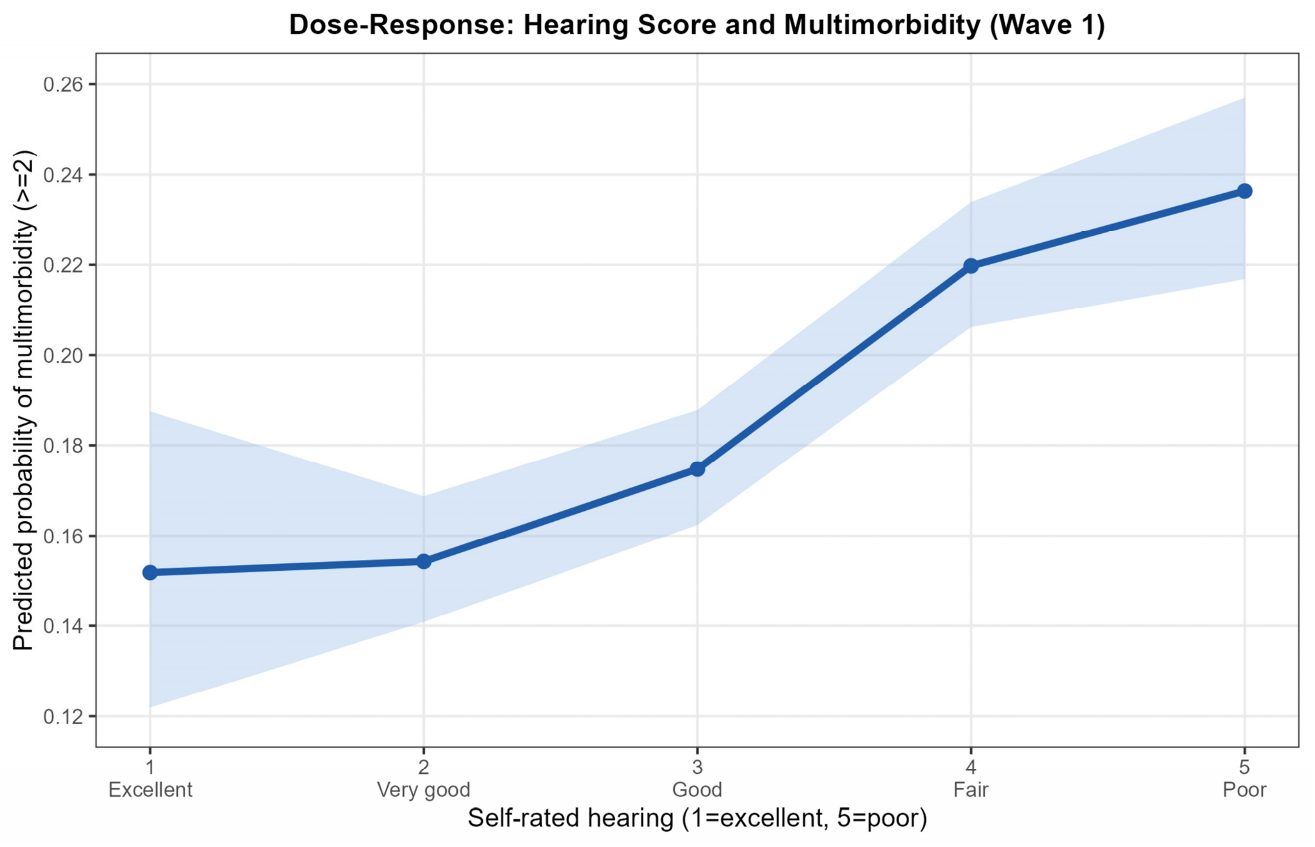

Supplementary Figure S3. Restricted cubic spline dose–response curve between self-rated hearing score and chronic disease multimorbidity. Predicted probability of multimorbidity increased from 15.2% at the best hearing score to 23.6% at the worst, with modest departure from linearity (likelihood-ratio test for non-linearity, P = 0.036).

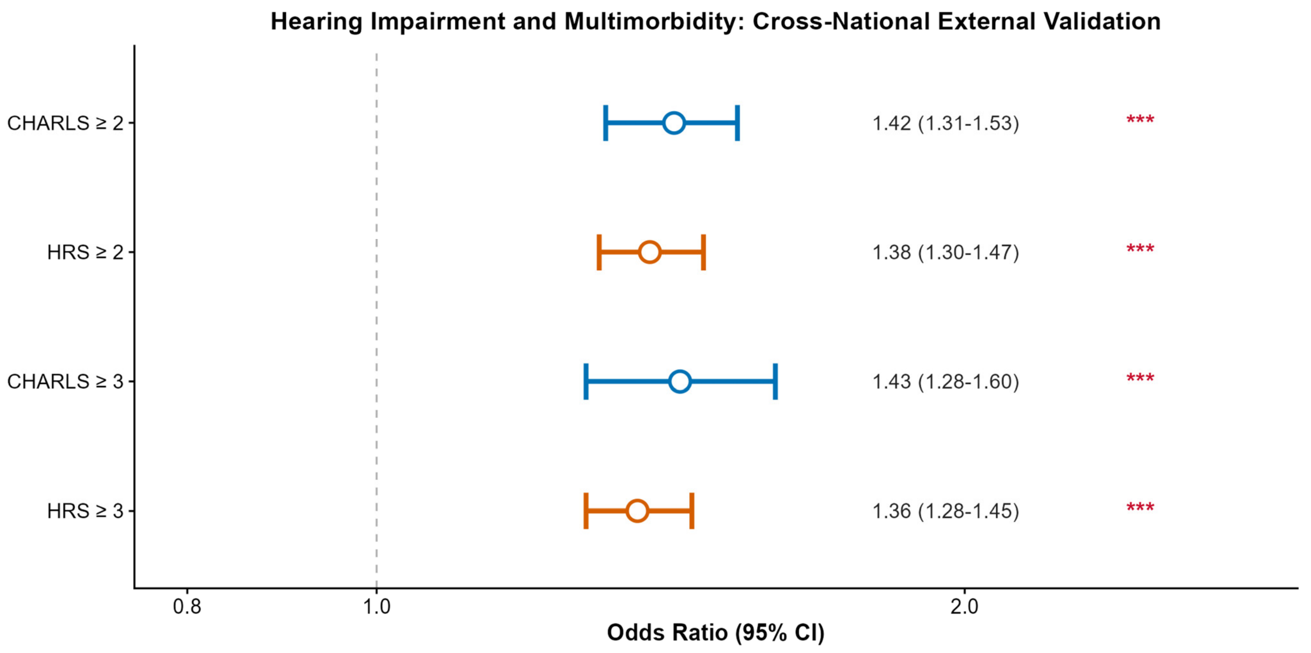

Supplementary Figure S4. HRS external validation of the hearing impairment-multimorbidity association. CHARLS Wave 1 (China) and HRS pooled waves 2010-2014 (USA) results are shown for

two or more and three or more chronic conditions. Multimorbidity in HRS was based on the seven chronic conditions shared with CHARLS. Error bars represent 95% confidence intervals.

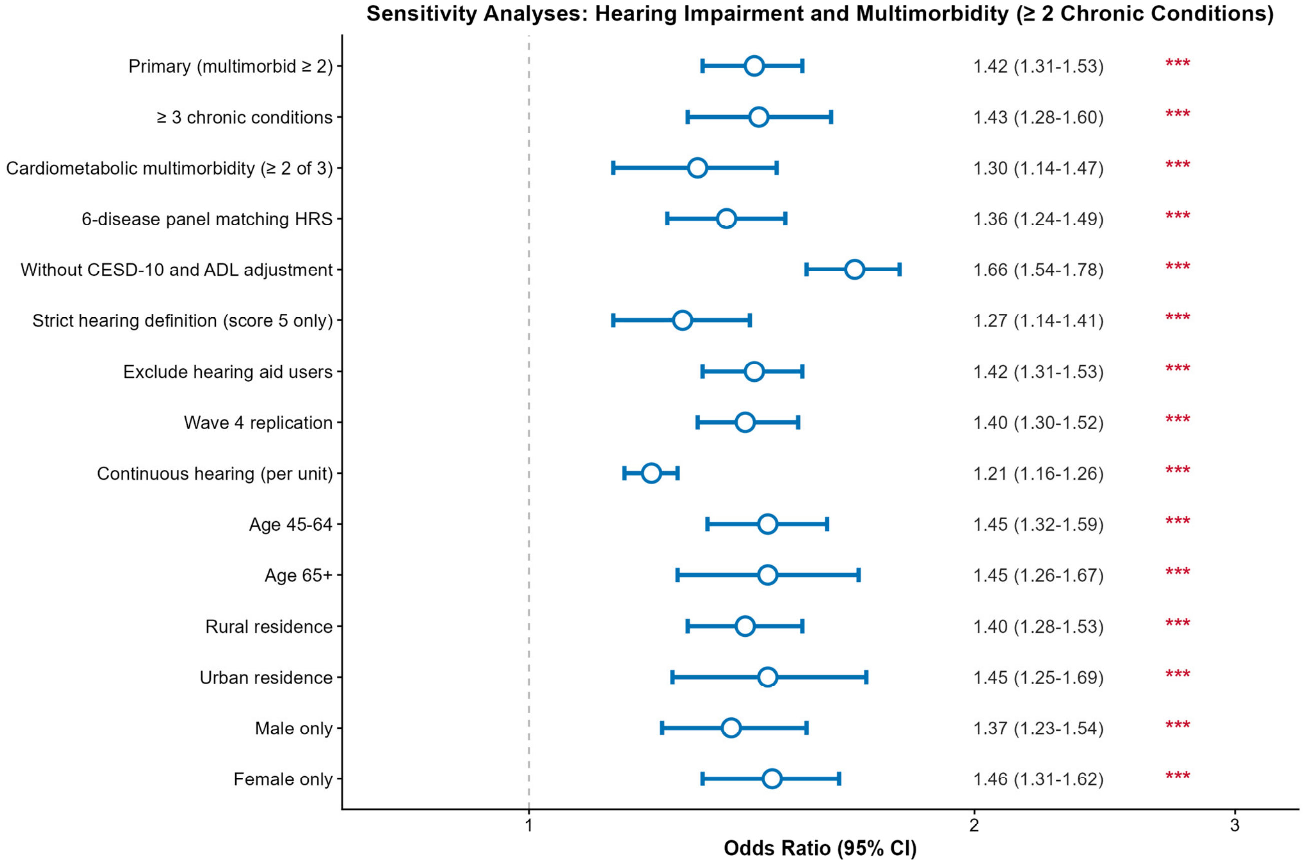

Supplementary Figure S5. Sensitivity analysis caterpillar plot for the association between hearing impairment and multimorbidity (two or more chronic conditions). Each row represents a different analytic specification. The primary analysis uses binary hearing impairment (scores 4-5); the continuous hearing score represents per-unit increase on the 1-5 scale. All models were fully adjusted for age, sex, education, urban/rural residence, CESD-10, and ADL disability, with cluster-robust standard errors.

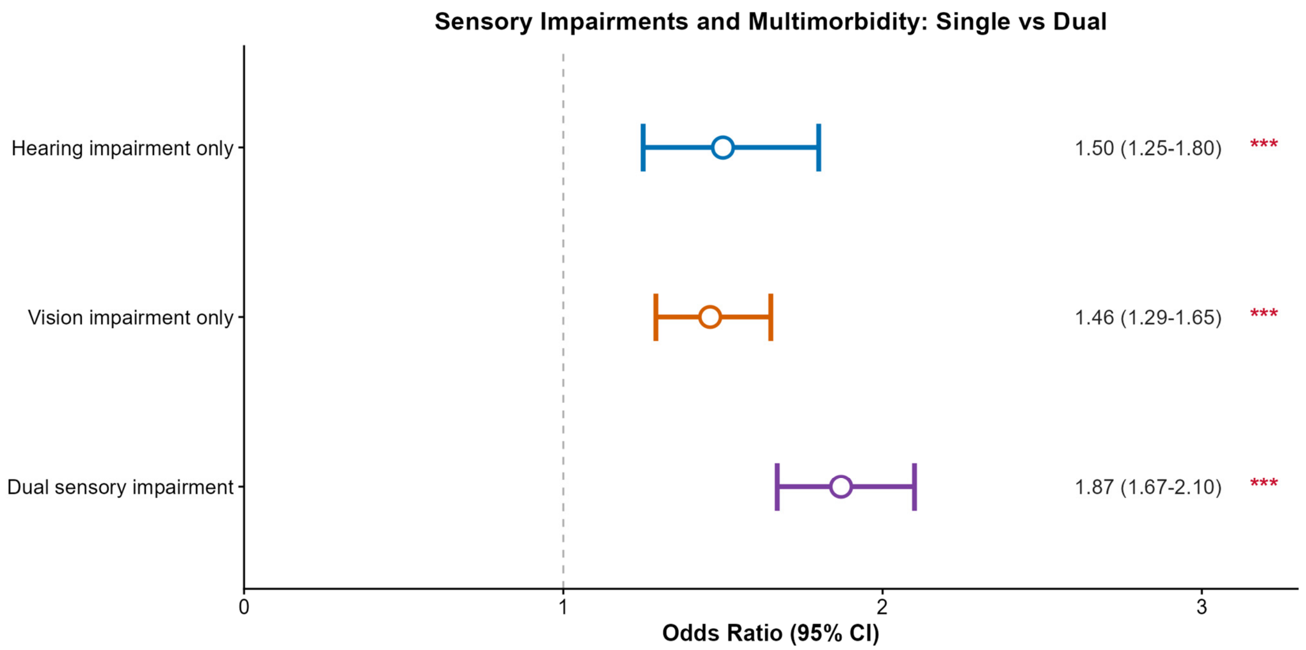

Supplementary Figure S6. Association between single versus dual sensory impairment and chronic disease multimorbidity (supplementary analysis). Hearing impairment only (OR = 1.50, 95% CI 1.25-1.80), vision impairment only (OR = 1.46, 95% CI 1.29-1.65), and dual sensory impairment (OR = 1.87, 95% CI 1.67-2.10), each relative to no sensory impairment. Models were fully adjusted. Error bars represent 95% confidence intervals.

Supplementary Tables

Supplementary Table S1. Sample characteristics by CHARLS wave

Wave	N	Hearing loss, %	Edentulism, %	With chronic disease data, n	Mean chronic conditions	Note
2011 (Wave 1)	17,594	54.2	8.6	17,573	1.04	Primary analysis wave; 99.9% disease data complete
2013 (Wave 2)	18,448	59.5	5.5	2,766	0.99	Disease items only for new respondents (14.9%); trajectory only
2015 (Wave 3)	20,965	63.1	4.3	529	0.96	Disease items only for new respondents (2.5%); trajectory only
2018 (Wave 4)	19,752	65.0	5.3	19,752	1.65	Replication wave; zdiagnosed carry-forward used

Values are n (%) or mean unless indicated. Waves 2-3 chronic disease items were administered exclusively to new respondents; therefore, cross-sectional multimorbidity analyses used Wave 1, and Wave 4 (2018) replication used the official carry-forward variables.

Supplementary Table S2. Missing data patterns (Wave 1)

Variable	Missing, n	Total, N	Missing, %
hearing_imp	37	17,594	0.2
edentulism	28	17,594	0.2

Variable	Missing, n	Total, N	Missing, %
n_chronic	21	17,594	0.1
cesd10	1,727	17,594	9.8
adl_disability	0	17,594	0.0
age	7	17,594	0.0
sex	0	17,594	0.0
edu_cat	22	17,594	0.1
urban_rural	18	17,594	0.1

Abbreviations: n, number. Missingness is reported for the Wave 1 analytic sample.

Supplementary Table S3. Association between sensory/oral impairments and individual chronic diseases

Disease	Exposure	OR (95% CI)	P value	N	Cases (n)
Hypertension	Hearing impairment	1.07 (1.01-1.15)	0.030	25,850	5,509
Hypertension	Edentulism	0.91 (0.81-1.03)	0.137	25,850	5,509
Dyslipidemia	Hearing impairment	1.25 (1.15-1.36)	<0.001	27,293	2,836
Dyslipidemia	Edentulism	0.84 (0.71-0.99)	0.033	27,293	2,836
Diabetes	Hearing impairment	1.17 (1.05-1.31)	0.004	28,134	1,658
Diabetes	Edentulism	0.93 (0.77-1.13)	0.496	28,134	1,658
Cancer	Hearing impairment	1.14 (0.89-1.45)	0.293	29,070	328
Cancer	Edentulism	1.19 (0.80-1.77)	0.397	29,070	328
Chronic lung disease	Hearing impairment	1.29 (1.17-1.42)	<0.001	28,058	2,347
Chronic lung disease	Edentulism	1.28 (1.11-1.49)	0.001	28,058	2,347
Kidney disease	Hearing impairment	1.40 (1.22-1.61)	<0.001	28,652	1,073
Kidney disease	Edentulism	1.05 (0.82-1.33)	0.707	28,652	1,073
Heart disease	Hearing impairment	1.33 (1.22-1.45)	<0.001	27,504	2,905
Heart disease	Edentulism	1.09 (0.94-1.26)	0.267	27,504	2,905
Stroke	Hearing impairment	1.20 (1.03-1.39)	0.018	28,975	956

Disease	Exposure	OR (95% CI)	P value	N	Cases (n)
Stroke	Edentulism	0.88 (0.69-1.11)	0.279	28,975	956
Arthritis	Hearing impairment	1.26 (1.18-1.34)	<0.001	25,195	6,512
Arthritis	Edentulism	1.30 (1.16-1.45)	<0.001	25,195	6,512

Abbreviations: OR, odds ratio; CI, confidence interval; N, number of participants; Cases, number with the individual condition. Analysis used pooled data across Waves 1-4 to maximize power. All models were fully adjusted for age, sex, education, urban/rural residence, CESD-10, and ADL disability, with cluster-robust standard errors. These individual-disease analyses were exploratory and P values were not adjusted for multiplicity.

Supplementary Table S4. Wave 4 (2018) replication analysis

Analysis	OR	95% CI	P value	N
Hearing -> multimorb >=2	1.40	1.30-1.52	<0.001	12,367
Hearing -> multimorb >=3	1.50	1.36-1.65	<0.001	12,367
Edentulism -> multimorb >=2	0.92	0.78-1.09	0.349	12,367
Edentulism -> multimorb >=3	0.95	0.79-1.14	0.583	12,367

Abbreviations: OR, odds ratio; CI, confidence interval; N, number of participants. Chronic disease status for previously diagnosed respondents was carried forward from the official carry-forward variables. The hearing-multimorbidity association was replicated in Wave 4 (OR 1.40 for two or more and 1.50 for three or more chronic diseases).

Supplementary Table S5. LCMM trajectory heterogeneity sensitivity analysis (model fit and convergence)

Specification	Single-class BIC	Single-class log-likelihood	Two-class BIC	Two-class converged	Convergence note
Hearing: random slope+intercept (primary)	65,872	-32,862	(did not converge)	No	ng=2-5 produced singular Hessian matrices or non-positive-definite variance-covariance under all attempted random-start specifications
Hearing: random intercept only (sensitivity)	65,864	-32,857	63,130	Yes	Converged with B starting values from the ng=1 fit
Edentulism: random slope+intercept (primary)	-3,736	2,102	(did not converge)	No	ng=2-5 produced singular Hessian matrices under all attempted specifications
Edentulism: random intercept only (sensitivity)	-3,736	2,102	-21,846	Yes	Converged with B starting values from the ng=1 fit

Abbreviations: LCMM, latent class linear mixed model; BIC, Bayesian Information Criterion. Under the primary random slope + intercept specification, models with two to five latent classes did not converge; the single-class model (hearing BIC = 65,872; edentulism BIC = -3,736) was the best-fitting

and only estimable solution. Under the simplified random-intercept specification, a two-class solution converged for hearing impairment, with classes distinguished primarily by baseline burden.

Supplementary Table S6. Wave 1 self-reported hearing impairment and edentulism predicting incident multimorbidity at Wave 4 (2018), CHARLS.

Sample	Outcome	Model	OR	95% CI	P	N
Baseline multimorbidity-free (<2 diseases)	Incident multimorbidity (>=2)	Model 1 (age, sex)	1.35	1.24-1.47	<0.001	9936
Baseline multimorbidity-free (<2 diseases)	Incident multimorbidity (>=2)	Model 2 (+ education, urban/rural)	1.36	1.25-1.48	<0.001	9936
Baseline multimorbidity-free (<2 diseases)	Incident multimorbidity (>=2)	Model 3 (+ CESD-10, ADL)	1.28	1.17-1.39	<0.001	9109
Baseline multimorbidity-free (CESD-eligible subsample)	Incident multimorbidity (>=2)	Model 2 (no mediator adjustment)	1.38	1.26-1.50	<0.001	9109
**Baseline multimorbidity-free (CESD-eligible subsample)**	**Incident multimorbidity (>=2)**	**Model 3 (full adjustment)**	**1.28**	**1.17-1.39**	**<0.001**	**9109**
Baseline multimorbidity-free (<2 diseases)	Incident multimorbidity (>=3)	Model 1 (age, sex)	1.35	1.21-1.51	<0.001	9936
Baseline multimorbidity-free (<2 diseases)	Incident multimorbidity (>=3)	Model 2 (+ education, urban/rural)	1.38	1.24-1.54	<0.001	9936

Baseline multimorbidity-free (<2 diseases)	Incident multimorbidity (>=3)	Model 3 (+ CESD-10, ADL)	1.28	1.14-1.44	<0.001	9109
Baseline multimorbidity-free (CESD-eligible sub-sample)	Incident multimorbidity (>=3)	Model 2 (no mediator adjustment)	1.40	1.25-1.57	<0.001	9109
**Baseline multimorbidity-free (CESD-eligible sub-sample)**	**Incident multimorbidity (>=3)**	**Model 3 (full adjustment)**	**1.28**	**1.14-1.44**	**<0.001**	**9109**
Baseline disease-free (0 diseases)	Incident multimorbidity (>=2)	Model 1 (age, sex)	1.35	1.19-1.53	<0.001	5576
Baseline disease-free (0 diseases)	Incident multimorbidity (>=2)	Model 2 (+ education, urban/rural)	1.36	1.20-1.53	<0.001	5576
Baseline disease-free (0 diseases)	Incident multimorbidity (>=2)	Model 3 (+ CESD-10, ADL)	1.30	1.14-1.48	<0.001	5057
Baseline disease-free (CESD-eligible sub-sample)	Incident multimorbidity (>=2)	Model 2 (no mediator adjustment)	1.40	1.23-1.59	<0.001	5057
**Baseline disease-free (CESD-eligible sub-sample)**	**Incident multimorbidity (>=2)**	**Model 3 (full adjustment)**	**1.30**	**1.14-1.48**	**<0.001**	**5057**

Baseline disease-free (0 diseases)	Incident multimorbidity (>=3)	Model 1 (age, sex)	1.50	1.26-1.79	<0.001	5576
Baseline disease-free (0 diseases)	Incident multimorbidity (>=3)	Model 2 (+ education, urban/rural)	1.52	1.27-1.82	<0.001	5576
Baseline disease-free (0 diseases)	Incident multimorbidity (>=3)	Model 3 (+ CESD-10, ADL)	1.41	1.17-1.71	<0.001	5057
Baseline disease-free (CESD-eligible subsample)	Incident multimorbidity (>=3)	Model 2 (no mediator adjustment)	1.56	1.29-1.88	<0.001	5057
**Baseline disease-free (CESD-eligible subsample)**	**Incident multimorbidity (>=3)**	**Model 3 (full adjustment)**	**1.41**	**1.17-1.71**	**<0.001**	**5057**
**Baseline multimorbidity-free - edentulism exposure**	**Incident multimorbidity (>=2)**	**Model 3 (full adjustment)**	**0.95**	**0.79-1.13**	**0.559**	**9109**
**Baseline multimorbidity-free - edentulism exposure**	**Incident multimorbidity (>=3)**	**Model 3 (full adjustment)**	**0.94**	**0.75-1.18**	**0.605**	**9109**

All models use cluster-robust standard errors by individual ID. Outcome = incident multimorbidity at Wave 4 among Wave 1 respondents free of multimorbidity at baseline. Sequential models: Model 1 adjusts age and sex; Model 2 additionally adjusts education and urban/rural residence; Model 3 additionally adjusts CESD-10 and ADL disability. Rows marked 'CESD-eligible subsample' restrict to respondents with complete CESD-10/ADL data and present Model 2 (without mediators) and Model 3 (with mediators) to isolate the effect of the potential mediators; comparison of M2 and M3 shows partial attenuation, consistent with partial mediation via depression/disability. Edentulism rows show the fully adjusted estimate for baseline edentulism; no significant association was observed.

Longitudinal results remain consistent after full adjustment: self-reported hearing impairment at Wave 1 predicts incident multimorbidity at Wave 4 (fully adjusted OR 1.28; 95% CI 1.17-1.39).

Supplementary Table S7. Harmonization of chronic disease definitions between CHARLS and HRS.

**Condition**	**CHARLS item**	**HRS item**	**Coding**	**Longitudinal handling**
Hypertension	DA007_1: diagnosed by a doctor	JNT003: doctor ever told high blood pressure	1 = yes	Carry-forward once diagnosed
Dyslipidemia	DA007_2: diagnosed by a doctor	JB044: high cholesterol	1 = yes	Carry-forward
Diabetes	DA007_3: diagnosed by a doctor	JB027: diabetes	1 = yes	Carry-forward
Cancer	DA007_4: diagnosed by a doctor	JB035: cancer	1 = yes	Carry-forward
Chronic lung disease	DA007_5 (COPD/asthma/chronic bronchitis)	NC028	1 = yes	Carry-forward
Kidney disease	DA007_9: diagnosed by a doctor	JB036: kidney disease	1 = yes	Carry-forward
Heart disease	DA007_7 (MI/CHD/angina/CHF)	JB010-JB015	1 = yes to any	Carry-forward
Stroke	DA007_8: diagnosed by a doctor	JB023: stroke	1 = yes	Carry-forward
Arthritis	DA007_13 (rheumatoid arthritis/rheumatism)	JB042 (arthritis/rheumatism)	1 = yes	Carry-forward

Both surveys assess the same nine physician-diagnosed chronic conditions via self-report. Each condition used an identical definition (self-report of a doctor's diagnosis) and identical coding (1 = yes) in both cohorts. In CHARLS, once a condition is reported, later waves carry the diagnosis forward (zdiagnosed flag in Wave 4); the same carry-forward principle applies in HRS. Exposure harmonization: CHARLS DA039 (5-level self-rated hearing) maps to HRS JNT025 (also 5-level); 'fair/poor' = impairment in both cohorts. Edentulism defined identically as self-report of having lost all natural teeth.

Supplementary Table S8. HRS external validation

Cohort	N	Hearing loss, %	Multimorbidity (≥2), %	OR (95% CI), ≥2 conditions	OR (95% CI), ≥3 conditions	RR (95% CI)
CHARLS Wave 1 (China)	15,842	54.2	28.0	1.42 (1.31-1.53)	1.43 (1.28-1.60)	1.207 (1.165-1.250)
HRS pooled 2010-2014 (USA)	60,525	21.6	58.9	1.38 (1.30-1.47)	1.36 (1.28-1.45)	1.106 (1.089-1.125)

Abbreviations: HRS, Health and Retirement Study; OR, odds ratio; CI, confidence interval; RR, relative risk. HRS (US): pooled waves 2010-2014, self-reported fair/poor hearing, same analytic approach as CHARLS, with multimorbidity based on seven shared chronic conditions. Consistent positive associations across two independent national cohorts.

Supplementary Table S9. Sensitivity and subgroup analyses

Analysis	OR	95% CI	P value	N
Strict hearing (score=5 only)	1.27	1.14-1.41	<0.001	15,842
Without CESD-10 and ADL adjustment	1.66	1.54-1.78	<0.001	15,842
Sex: male	1.37	1.23-1.54	<0.001	7,432
Sex: female	1.46	1.31-1.62	<0.001	8,410
Age: 45-64	1.45	1.32-1.59	<0.001	11,585
Age: 65+	1.45	1.26-1.67	<0.001	4,257
Residence: rural	1.40	1.28-1.53	<0.001	12,267
Residence: urban	1.45	1.25-1.69	<0.001	3,575
Cardiometabolic multimorbidity (>=2 of 3)	1.30	1.14-1.47	<0.001	15,595
Hearing x Edentulism interaction	0.78	0.60-1.03	0.082	15,842
Exclude hearing aid users	1.42	1.31-1.53	<0.001	15,764
Hearing impairment only (vs none)	1.50	1.25-1.80	<0.001	15,842
Vision impairment only (vs none)	1.46	1.29-1.65	<0.001	15,842
Dual sensory impairment (vs none)	1.87	1.67-2.10	<0.001	15,842
6-disease panel matching HRS (>=2 of 6)	1.36	1.24-1.49	<0.001	15,842

Abbreviations: OR, odds ratio; CI, confidence interval; N, number of participants. Unless indicated, all models were fully adjusted for age, sex, education, urban/rural residence, CESD-10, and ADL disability, with cluster-robust standard errors.
